# Dengue Infections during COVID-19 Period: Reflection of Reality or Elusive Data Due to Effect of Pandemic

**DOI:** 10.3390/ijerph191710768

**Published:** 2022-08-29

**Authors:** Sakirul Khan, Sheikh Mohammad Fazle Akbar, Takaaki Yahiro, Mamun Al Mahtab, Kazunori Kimitsuki, Takehiro Hashimoto, Akira Nishizono

**Affiliations:** 1 Department of Microbiology, Faculty of Medicine, Oita University, Yufu 879-5593, Oita, Japan; 2 Department of Gastroenterology and Metabology, Ehime University Graduate School of Medicine, Toon 791-0295, Ehime, Japan; 3 Miyakawa Memorial Research Foundation, Tokyo 107-0062, Japan; 4 Research Center for Global and Local Infectious Diseases, Faculty of Medicine, Oita University, Yufu 879-5593, Oita, Japan; 5 Division of Interventional Hepatology, Bangabandhu Sheikh Mujib Medical University, Dhaka 1000, Bangladesh; 6 Infection Control Center, Oita University Hospital, Yufu 879-5593, Oita, Japan

**Keywords:** COVID-19 pandemic, dengue, sporadic increase, practical containment, Asia, Latin America

## Abstract

The outbreak of coronavirus disease 2019 (COVID-19) devastated the overall health management strategy of most countries. In this scenario, the present study provided insights into the possible impact of the COVID-19 pandemic on dengue infection. This ecological study retrieved data from WHO/Government reporting system from 22 major dengue epidemic countries. Incidence of dengue infections during the pre-COVID-19 time (2015~2019) and COVID-19 period (2020~2021) was compared. A correlation between the dengue and COVID-19 cases and predicted dengue incidence in 2022 was calculated using the linear regression equation. Data indicated that dengue incidences across the studied area decreased by 16% during the pandemic period (2.73 million vs. 2.29 million; *p* < 0.05) than the same reported in pre-COVID-19 time. Although countries in Latin America reported more cases than Asia, a positive correlation (r = 0.83) between dengue and COVID-19 cases was observed in Asia. Prediction analysis warned that specific preparation for dengue management is needed in some countries of both regions in 2022 to contain the upsurge in incidences. Due to the similar nature of symptoms of dengue and COVID-19, a state of confusion will be prevailing during the ongoing pandemic. Therefore, comprehensive and evidence-based scientific approaches were warranted at all levels.

## 1. Introduction

Dengue is an emerging mosquito-borne viral disease resulting in approximately 390 million estimated cases globally every year and remains the world’s fastest-growing tropical infectious disease [1,2]. Most of dengue infections remain unnoticed or unreported making it a complex public health problem. Even then, the World Health Organization (WHO) has mentioned an 8-fold increase of reported dengue cases during the last two decades (from 505,430 cases in 2000 to over 2.4 million in 2010, and 5.2 million in 2019) [3]. The number of confirmed death has been increased from 960 in 2000 to 4032 in 2015 [3]. Deaths attributable to dengue increased to 36,055 in 2019 [4]. Under this prevailing situation of the ongoing dengue epidemic, the pandemic of coronavirus disease 2019 (COVID-19), caused by severe acute respiratory syndrome coronavirus 2 (SARS-CoV-2), has shattered the health care delivery system of most the countries during last 2 and half years with reported cases of COVID-19 of more than 550 million and COVID-19-related deaths reached to 6.3 million [5].

Accordingly, as of today, many countries are struggling with one serious pandemic (COVID-19) and one progressing epidemic (dengue). Incidentally, these two pathological processes exhibit several overlapping symptoms that make it difficult to establish the causative relation [6,7]. It is also true that the co-infection of dengue and SARS-CoV-2 is a reality [8]. Several studies have shown some possible impacts of dengue on SARS-CoV-2 and vice versa [9,10,11]. For downplaying the spread of SARS-CoV-2, strict public health measures that have been extended from complete lock-down to implementation of several public health measures have been recommended by the WHO and implemented by almost all countries of the world. However, the reality indicates that due to the inherent nature of SARS-CoV-2 and the emergence of mutations and variants, control of COVID-19 may be a time-consuming endeavor for the world population. In addition to the COVID-19-related unprecedented situation, dengue would retain its epidemic status in most resource-constrained countries of Asia and Latin America. Indeed, control measures of dengue infection are neglected in many epidemic countries as excessive prioritization is given to preventing and control of SARS-CoV-2 infections [12,13].

These scenarios indicate that there should be a reasonable understanding of the dengue epidemic so that various public health programs may be validated with yet unknown kinetics of COVID-19 in the global paradigm. As continuous surveillance is a critical component of dengue prevention and control programs, in this study, we have analyzed the incidences of dengue during the COVID-19 pandemic period in various dengue-prone countries of Asia and Latin America to address some concerns about dengue infections.

## 2. Materials and Methods

### 2.1. Study Design and Geographical Area

This ecological study was conducted by using dengue and COVID-19 data retrieved from WHO/Government or reference reporting system. The study area covers 22 major dengue epidemic countries [11 from Asia (Philippines, Vietnam, India, Indonesia, Malaysia, Thailand, Sri Lanka, Bangladesh, Singapore, Pakistan, and Nepal), and 11 from Latin America (Brazil, Mexico, Nicaragua, Colombia, Paraguay, Peru, Honduras, Bolivia, Argentina, Ecuador, and Venezuela)] that located between 30° N and 30° S. Most of the selected countries are also severely affected by SARS-CoV-2 during the pandemic period [5].

### 2.2. Data Collection and Ethical Aspects

A systematic data search for dengue and COVID-19 incidences was conducted in June 2022. Data for dengue infections, COVID-19 cases, and the total population of each country were extracted from the official (WHO/Government) or reference reporting system [14,15,16,17,18,19,20,21]. The database contains updated epidemiological information necessary to understand the incidence of these two infectious diseases. After data extractions, at least two authors cross-checked the data to ensure consistency. Any disagreement that appeared during data extraction was resolved by group consensus.

This study was not submitted to any ethical body for clearance as we used only secondary aggregate data obtained from the public domain that does not allow for the identification of the cases assessed.

### 2.3. Data Analysis

We conducted a descriptive analysis of the dengue and COVID-19 incidences during the study period (for dengue: from January 2015~December 2021; for COVID-19: from January 2020~December 2021). Several databases were constructed using Microsoft^®^ Excel (Redmond, WA, USA) for each country located in Asia and Latin America. The date of notification of cases was taken into account to determine the epidemiological years of dengue and COVID-19. The dengue cases were regarded as a variable consisting of two values, which allowed for the division of time into two stages: pre-COVID-19 time (2015~2019) and during the COVID-19 period (2020–2021). Incidences per million population were calculated based on the variable “dengue or the COVID-19 cases per epidemiological year in each country” and taking into account the total population of the country. Student’s test-test was used to analyze the percentage of change in the average dengue incidences at the pre-COVID-19 time and COVID-19 pandemic period in the countries of Asia and Latin America. To clarify any correlations that might exist between dengue cases and the COVID-19 cases, the Pearson correlation test was performed for each region or both regions. Predicted dengue incidence in 2022 was calculated based on the reported cases between 2015~2021 by using the linear regression equation *y* = *a* + *bx*, where the *a* constant (intercept) is $a=\bar{y}-b\bar{x}$, and the *b* coefficient (slope of the line) is $b=\frac{\Sigma \left(x-\bar{x}\right)\left(y-\bar{y}\right)}{\Sigma {\left(x-\bar{x}\right)}^{2}}$. The values of $\bar{x}$ and $\bar{y}$ are the sample means (averages) of the known *x*-values and *y*-values. The analyses were performed using Microsoft^®^ Excel and SAS, version 9.4 (Cary, NC, USA). The statistical significance was set at *p* < 0.05.

## 3. Results

### 3.1. Dengue Infections during the Pre-COVID-19 (2015~2019) and COVID-19 (2020~2021) Period

**Figure 1:**
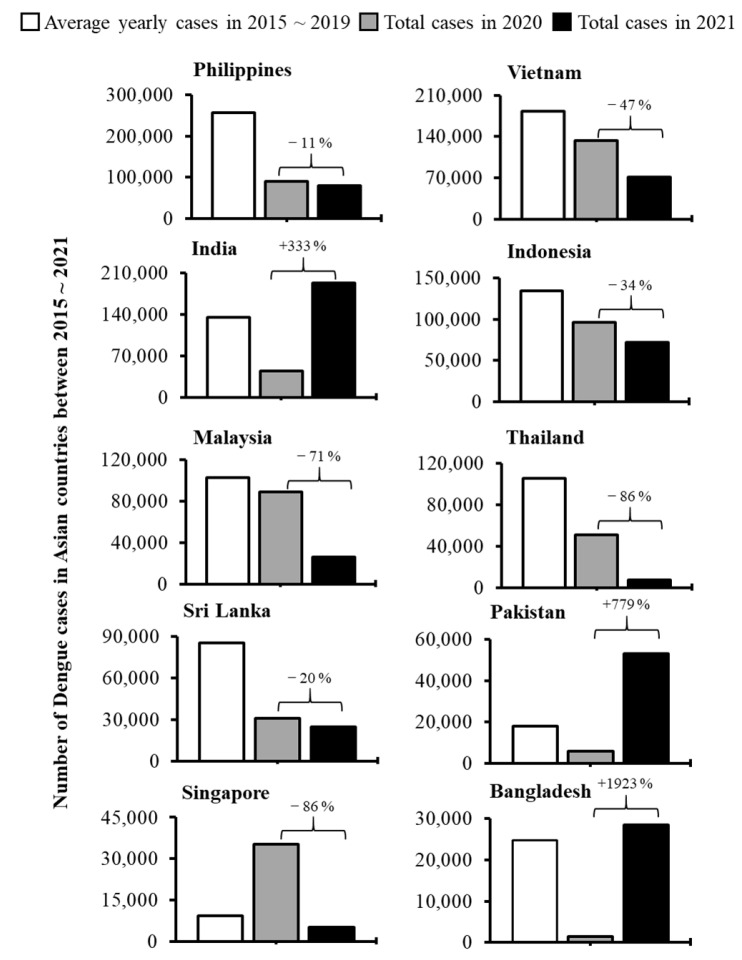
Comparison of dengue incidences in major dengue epidemic countries of Asia between the pre-COVID-19 time (2015~2019) and COVID-19 period (2020~2021).

**Figure 2:**
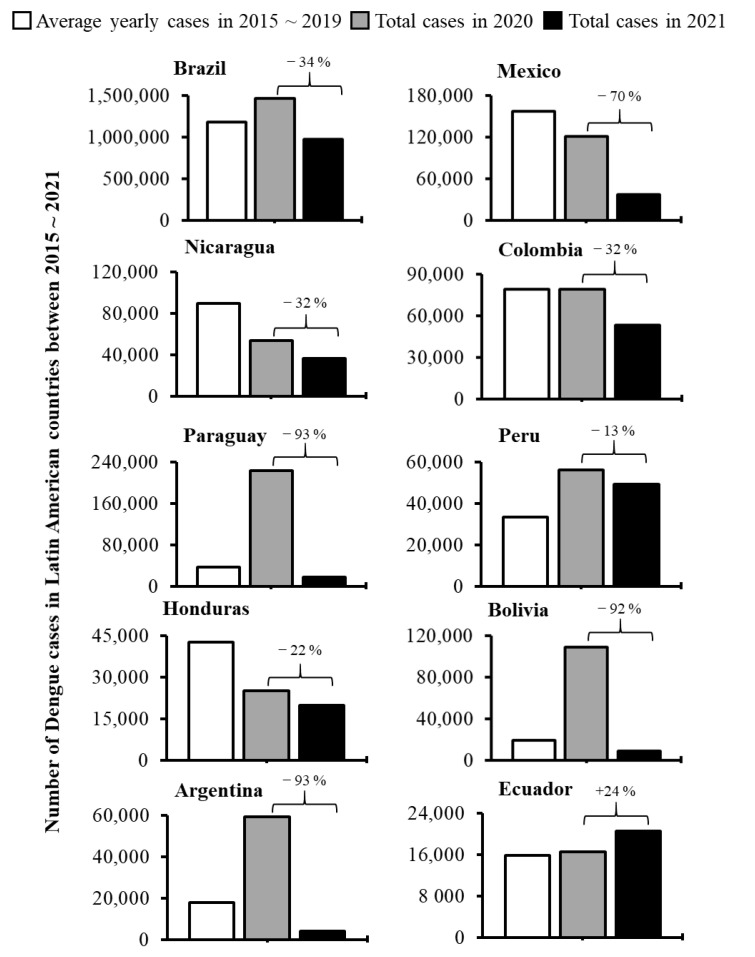
Comparison of dengue incidences in major dengue epidemic countries of Latin America between the pre-COVID-19 time (2015~2019) and COVID-19 period (2020~2021).

**Figure 3:**
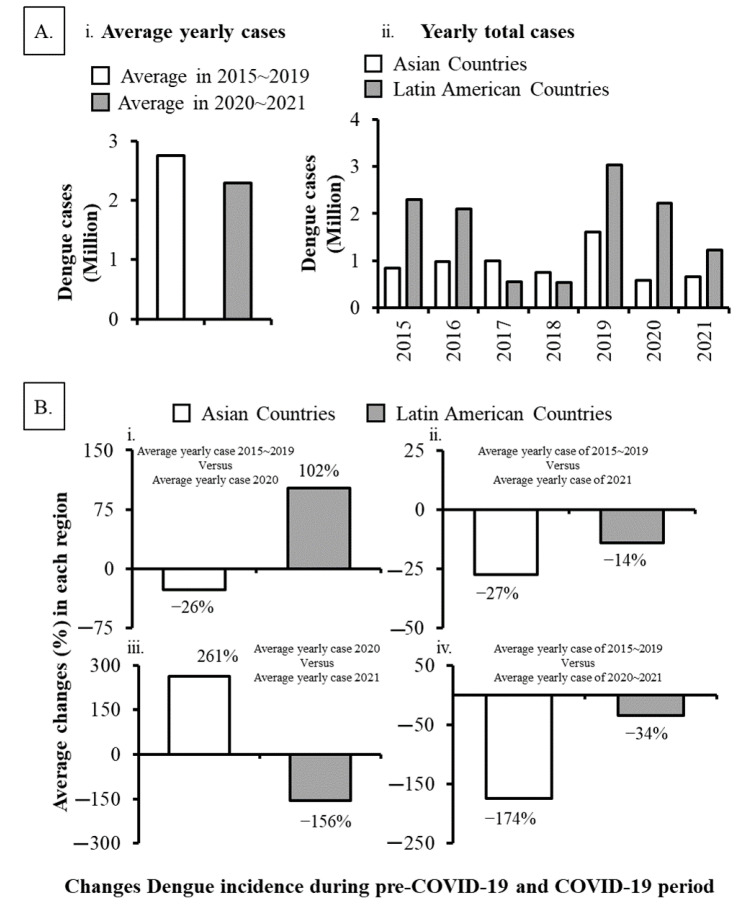
Dengue infection patterns in Asia verse Latin America during the pre-COVID-19 time (2015~2019) and COVID-19 period (2020~2021). (**A**) (**i**) Average dengue incidences in both regions during the pre-COVID-19 time and COVID-19 period. (**ii**) Epidemiological diversities of dengue incidences from year to year in Asia and Latin America from 2015 to 2021. (**B**) (**i**−**iv**) Average change of dengue incidences in each region during the pre-COVID-19 time and COVID-19 period.

To assess the effects of COVID-19 on dengue infections, we analyzed dengue cases during the pre-COVID-19 time and COVID-19 period in the countries of Asia (Figure 1) and Latin America (Figure 2). Out of 22 major dengue epidemic countries considered for this study, only 7 countries (Brazil, Peru, Bolivia, Ecuador, Paraguay, Argentina, and Singapore) reported an increased number of dengue incidence during 1st year of the COVID-19 pandemic period (in 2020) comparing with the average cases reported during the pre-COVID-19 time (2015~2019). When we compared the incidences within the pandemic years, 3 of 22 countries (Bangladesh, Pakistan, and India) reported a remarkable increase of dengue cases during the 2nd year of the COVID-19 pandemic (in 2021) than those accounted for 1st year of the pandemic (in 2020) (Figure 1 and Figure 2). In fact, India reported over a 3-fold increase in incidence (44,585 vs. 193,245 cases), Pakistan reported a more than 7-fold increase (6016 vs. 52,894) while Bangladesh reported a sharp increase (over 19-fold; 1405 vs. 28,429) of dengue cases (Figure 1). Such a sharp increase in dengue cases was not observed in any other countries during the COVID-19 pandemic period (Figure 1 and Figure 2). Collectively, although the sporadic increase in dengue cases observed in some countries, overall the dengue incidences were decreased by 16% (2.73 million vs. 2.29 million; *p* < 0.05) across the study area during the 2 years of the COVID-19 pandemic compared to the average number accounted in 5-year of pre-COVID-19 time (Figure 3Ai).

### 3.2. Comparison of Dengue Infections between Asian and Latin American Countries

To get an impression of dengue infection patterns in Asia and Latin America, we compared the reported incidences between these two regions. Considering the epidemiological diversities from year to year, dengue has maintained an endemic status across the studied countries in Asia and Latin America (Figure 3Aii). When comparing the cases between regions, countries in Latin America reported more dengue cases than the countries in Asia for 5-year of pre-COVID-19 time (8.50 million vs. 5.16 million; *p* < 0.05) or 2-year of the COVID-19 pandemic period (3.45 million vs. 1.14 million; *p* < 0.05). During 1st year of the COVID-19 pandemic (in 2020, on an average 102% more dengue cases were reported in Latin American countries compared to the average incidence in 2015~2019 while it was decreased by 26% in Asian countries (Figure 3Bi). On the other hand, countries in both regions reported fewer dengue cases in the 2nd year of COVID-19 pandemic (in 2021) (−27% for Asia and −14% for Latin America; *p* > 0.05) (Figure 3Bii) than pre-COVID-19 time. Compared to the dengue cases between 2020 and 2021, countries in Asia reported more changes in average incidence than Latin Americas in 2021 (261% vs. −156%; *p* < 0.05) (Figure 3Biii). However, overall, the Asian region reported a lower number of dengue cases during the 2-year period of the COVID-19 pandemic (−174% vs. −34%; *p* < 0.05) than the average case reported in pre-COVID-19 time (Figure 3Biv).

### 3.3. Relationship between Dengue and COVID-19 Cases

**Figure 4:**
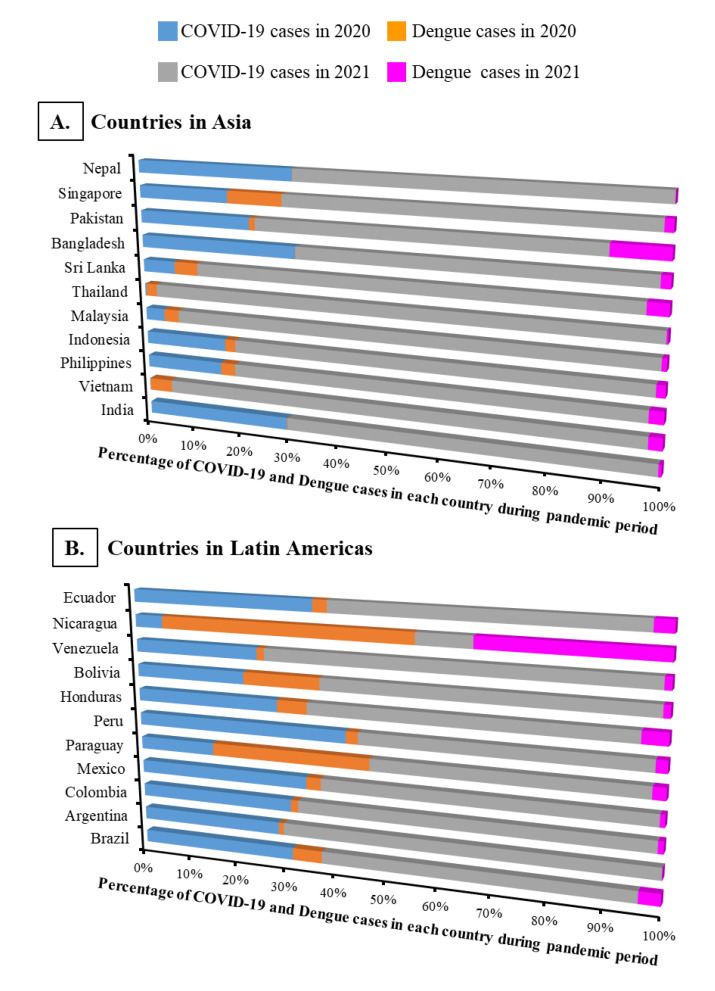
Country-wise percentage of dengue and COVID-19 incidences during the pandemic period (2020~2021). (**A**) Percentage of dengue and COVID-19 incidences in each country located in Asia. (**B**) Percentage of dengue and COVID-19 incidences in each country located in Latin America.

**Figure 5:**
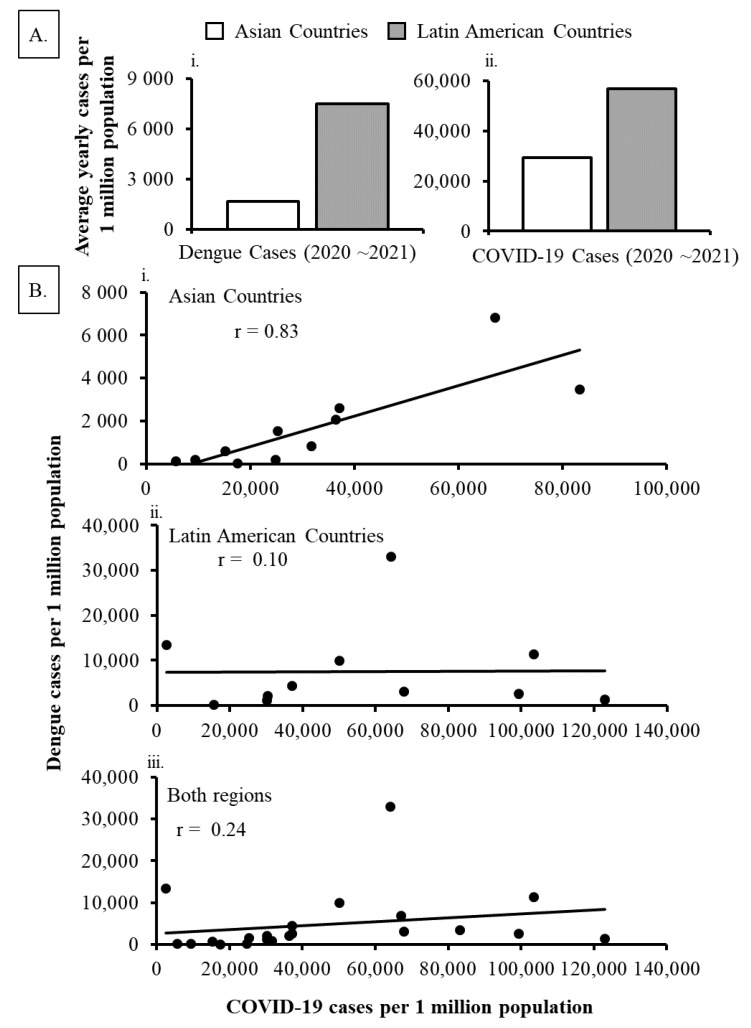
Dengue and COVID-19 cases per million population in Asia and Latin America during the pandemic period (2020~2021). (**A**) Average yearly (**i**) dengue and (**ii**) COVID-19 cases per million population. (**B**) Correlation between dengue and COVID-19 cases per million population of (**i**) Asia, (**ii**) Latin America or (**iii**) both regions.

Although dengue cases showed diversities in 2020 and 2021, a higher number of COVID-19 cases were observed in all studied countries in 2021 (Figure 4; Supplementary Table S1). When analyzing the percentage of COVID-19 and dengue cases in each country, only Nicaragua reported a higher percentage of dengue incidences than COVID-19 cases in both years of the pandemic (Figure 4B; Supplementary Table S1). Paraguay also reported more dengue cases than COVID-19 cases in 1st year of the pandemic (in 2020). Similar to dengue incidences, countries in Latin America reported more COVID-19 cases per 1 million population than in Asia (Figure 5Ai,ii). On the other hand, in contrast to Latin America, a strong positive correlation (r = 0.83) between dengue cases/million population and COVID-19 cases/million population was observed in the Asian countries (Figure 5Bi–iii). However, the correlation between dengue and COVID-19 cases did not show any specific pattern (decrease or increase) when countries were considered as a totality (r = 0.24).

### 3.4. Prediction of Dengue Incidences in 2022

Predicted analysis revealed that there will be no major changes in total dengue infections across the study area in 2022 although some exceptional upsurge will be observed as seen in the previous 2-year of the pandemic period (Supplementary Table S2). Notably, Bangladesh and Pakistan may be accounted for the double degree of dengue cases in 2022 compared to the average incidence reported between 2015~2021. Similarly, dengue cases may be increased in Bolivia, Paraguay, Singapore, and Nepal in 2022. On the other hand, Mexico, Ecuador, Argentina, Indonesia, Malaysia, Thailand, and Sri Lanka may report fewer dengue cases in 2022 than the average number reported in the past 7 years (Supplementary Table S2).

## 4. Discussion

The present study provided a comprehensive impression of global dengue infection patterns during the 2-year of COVID-19 pandemic period (2020~2021) by compiling data from 22 countries located in Asia and Latin America that harbor major dengue cases from a global perspective. Compared to the average cases observed during the pre-COVID-19 time (2015~2019), the overall dengue cases decreased by 16% during the COVID-19 pandemic period, although sporadic increases in incidences were observed in some countries (Figure 1, Figure 2 and Figure 3). However, irrespective of epidemiological diversities between 2020 and 2021, the overall decline of dengue cases was more pronounced in Asia than in Latin America although the Asian countries showed a positive correlation between dengue and COVID-19 cases (Figure 3, Figure 4 and Figure 5; Supplementary Table S1).

A recent study reported that restriction of COVID-19-related human movements is associated with the reduction of dengue cases during 1st year of the COVID-19 pandemic [22]. However, an upsurge of dengue cases is observed in Brazil, Peru, Bolivia, Paraguay, Argentina, and Singapore during 1st year of the COVID-19 pandemic time (Figure 2) although most of these countries maintain strong COVID-19-related measures during the early time of the pandemic. Similarly, dengue incidences sharply increased in Bangladesh, Pakistan, and India during 2nd year of the COVID-19 pandemic (in 2021) when these countries applied relatively strong COVID-19-related measures due to the severe outbreak of the deadly Delta variant of SARS-CoV-2 [23]. Therefore, it remains unclear whether strict COVID-19-related social measures or underreporting due to the overburden of management of COVID-19 are related to the decline of reported dengue cases during the pandemic period. However, regular observation and periodical analysis of dengue incidences will provide more accurate insights into this issue.

Similar to dengue infection patterns, divergent incidences of COVID-19 cases were reported in the studied countries. Since the COVID-19 pandemic began in Wuhan, China in December 2019, the virus spread all over the world within a few months. With the experience of the ongoing pandemic, some countries in both regions, notably Thailand, Vietnam, and Nicaragua reported only a few COVID-19 cases during the first year of the pandemic (Supplementary Table S1). The low cases of COVID-19 may coincide with the introduction of strict public health and social measures including school closer in some countries, particularly in Thailand [24]. Also, cultural activities and control measures (social distancing and use of face masks) in some countries may be useful for preventing the spread of SARS-CoV-2 in the early stage. However, in the second year of the COVID-19 pandemic when some countries relaxed COVID-19-related restrictions along with the emergence of deadly and highly transmittable variants of SARS-CoV-2, most of the studied countries reported a significant number of COVID-19 cases (Figure 4; Supplementary Table S1). Although the relationship between COVID-19 and dengue cases may be a complex phenomenon, our present study found a positive correlation (r = 0.83) between dengue cases/million population and COVID-19 cases/million population in Asian countries (Figure 5Bi).

The results of this study have some limitations. We have been formulating this article on the basis of data provided by WHO or different government agencies. The real picture of the incidence and prevalence of both SARS-CoV-2 and dengue viruses may be much more as similar facts have been reported in developing countries [25,26,27]. Moreover, dengue is prevalent in 129 countries [3], however, we used data from only 22 major dengue-affected countries. Therefore, the results of the present study may differ from global dengue infection patterns during the COVID-19 pandemic period. Also, we did not consider the environmental and social factors to predict dengue incidences during the ongoing pandemic year which may not provide the actual prediction data.

However, as of the end of June 2022, when this article is compiled, these countries have been reporting daily increases in dengue and COVID-19 cases with almost no approach to get insights into the underlying management policies to prevent the worst scenario. Many Latin American countries already reported more dengue cases than those recorded in 2021 [14]. Similar increasing patterns of dengue cases were also observed in several Asian countries even though dengue season just started in this region [15,16,17,18,19,20,28]. However, the epidemiological data in the middle of 2022 clearly shows that the worst may be yet to come as dengue infections have been increasing in prevailing countries and it will continue usually until November. Our prediction analysis also revealed that many countries in both regions may see more dengue cases during the ongoing pandemic year (Supplementary Table S2). Therefore, it is possible that an alarming situation will be continuing in many dengue epidemic countries due to the co-existence of one pandemic (COVID-19) and one epidemic (dengue) with similar and overlapping clinical symptoms. Such co-existing conditions may have serious consequences for both patient care and public health perspectives. Particularly, confusion remains in the diagnosis of COVID-19 and dengue because there is a possibility of cross-reactivity between dengue virus and SARS-CoV-2, which can lead to false-positive dengue serology among COVID-19 patients and vice versa [8,29]. Indeed, it is already known that patients with co-infection of these viruses follow a downhill course and fatal outcomes [8]. Moreover, dengue re-infection may present as a more severe disease due to antibody-dependent enhancement (ADE) [30]. Pieces of evidence have also suggested that SARS-CoV-2 is capable of inducing ADE [31] and many dengue patients may have co-infection with SARS-CoV-2. We are not sure at this moment if there is any role of SARS-CoV-2 and dengue virus in inducing a deadly infection at the cellular and molecular levels. However, this demands more insights into the role of the co-infection of these viruses that enhance ADE. Under these realities, the correct diagnosis and treatment of re-infection or co-infection of these two viruses pose a substantial challenge during the ongoing pandemic period due to overlapping clinical and laboratory parameters.

## 5. Conclusions

During 2-year of COVID-19 pandemic period, the overall dengue incidences decreased in major dengue epidemic countries when compared to the average cases reported in 5-year of pre-COVID-19 time. However, some exceptional sporadic upsurge was recorded in some countries, particularly in South Asia. A similar upsurge of dengue cases may be seen during the ongoing COVID-19 pandemic year (in 2022). Considering the serious condition of dengue during the COVID-19 pandemic, the present situation should be scientifically reviewed not only from the number of infected patients but also from the nature of the infections. Under these realities, comprehensive and evidence-based scientific programs at all levels such as epidemiology, pathogenesis, and management should be immediately planned with an eye on the strengths and weaknesses of health care delivery services of developing countries. As the world is moving through the emergency conditions with the double punch of one pandemic (COVID-19) and one epidemic (dengue), the solution of which would come from scientific works of international collaboration by various stakeholders like WHO and allied international institutions as the facilities are yet to optimize in resource-constrained developing dengue epidemic countries.

## Data Availability

The corresponding author had full access to all data in the study and had final responsibility for the decision to submit for publication. The data that support the findings of this study are available on request from the corresponding author [S.K.].

## Supplementary Materials

The following supporting information can be downloaded at: https://www.mdpi.com/article/10.3390/ijerph191710768/s1, Table S1: COVID-19 and dengue cases in the Asian and Latin American countries during the COVID-19 pandemic period (2020~2021); Table S2: The number of dengue cases may observe during the ongoing pandemic year (2022) in the Asian and Latin American countries.

## References

[1] Bhatt S., Gething P.W., Brady O.J., Messina J.P., Farlow A.W., Moyes C.L., Drake J.M., Brownstein J.S., Hoen A.G., Sankoh O. (2013). The global distribution and burden of dengue. Nature.

[2] Kularatne S.A., Dalugama C. (2022). Dengue infection: Global importance, immunopathology and management. Clin. Med..

[3] World Health Organization (WHO) Dengue and Severe Dengue (2022). https://www.who.int/news-room/fact-sheets/detail/dengue-and-severe-dengue#:~:text=Many%20cases%20are%20also%20misdiagnosed,with%20any%20severity%20of%20disease.

[4] Yang X., Quam M.B.M., Zhang T., Sang S. (2021). Global burden for dengue and the evolving pattern in the past 30 years. J. Travel Med..

[5] World Health Organization (WHO) Coronavirus (COVID-19) Dashboard. https://covid19.who.int/.

[6] Cardona-Ospina J.A., Arteaga-Livias K., Villamil-Gómez W.E., Pérez-Díaz C.E., Bonilla-Aldana D.K., Mondragon-Cardona Á., Solarte-Portilla M., Martinez E., Millan-Oñate J., López-Medina E. (2021). Dengue and COVID-19, overlapping epidemics? An analysis from Colombia. J. Med. Virol..

[7] Harapan H., Ryan M., Yohan B., Abidin R.S., Nainu F., Rakib A., Jahan I., Emran T.B., Ullah I., Panta K. (2021). COVID-19 and dengue: Double punches for dengue-endemic countries in Asia. Rev. Med. Virol..

[8] Tsheten T., Clements A.C.A., Gray D.J., Adhikary R.K., Wangdi K. (2021). Clinical features and outcomes of COVID-19 and dengue co-infection: A systematic review. BMC Infect. Dis..

[9] Liyanage P., Rocklöv J., Tissera H.A. (2021). The impact of COVID-19 lockdown on dengue transmission in Sri Lanka; A natural experiment for understanding the influence of human mobility. PLoS Negl. Trop. Dis..

[10] Lin S.F., Lai C.C., Chao C.M., Tang H.J. (2021). Impact of COVID-19 preventative measures on dengue infections in Taiwan. J. Med. Virol..

[11] Vicente C.R., Silva T.C.C.D., Pereira L.D., Miranda A.E. (2021). Impact of concurrent epidemics of dengue, chikungunya, zika, and COVID-19. Rev. Soc. Bras. Med. Trop..

[12] Olive M.M., Baldet T., Devillers J., Fite J., Paty M.-C., Paupy C., Quénel P., Quillery E., Raude J., Stahl J.-P. (2020). The COVID-19 pandemic should not jeopardize dengue control. PLoS Negl Trop Dis..

[13] Plasencia-Dueñas R., Failoc-Rojas V.E., Rodriguez-Morales A.J. (2022). Impact of the COVID-19 pandemic on the incidence of dengue fever in Peru. J. Med. Virol..

[14] Reported Cases of Dengue Fever in The Americas. The Pan American Health Organization (PAHO). https://www3.paho.org/data/index.php/en/mnu-topics/indicadores-dengue-en/dengue-nacional-en/252-dengue-pais-ano-en.html.

[15] World Health Organization (WHO) Dengue Situation Updates (2020). WHO Regional Office for the Western Pacific. https://apps.who.int/iris/handle/10665/330698.

[16] World Health Organization (WHO) Dengue Situation Updates (2021). WHO Regional Office for the Western Pacific. https://apps.who.int/iris/handle/10665/341149.

[17] Dengue Worldwide Overview European Centre for Disease Prevention and Control. https://www.ecdc.europa.eu/en/dengue-monthly.

[18] Daily Dengue Status Report Directorate General of Health Services, Bangladesh. https://old.dghs.gov.bd/index.php/bd/home/5200-daily-dengue-status-report.

[19] Dengue Situation in India National Center for Vector Borne Diseases Control (NCVBDC), Ministry of Health & Family Welfare, Government of India. https://nvbdcp.gov.in/index4.php?lang=1&level=0&linkid=431&lid=3715.

[20] Distribution of Notification Dengue Cases Epidemiology Unit, Ministry of Health, Sri Lanka. http://www.epid.gov.lk/web/index.php?Itemid=448.

[21] Worldometer Countries in the world by population. https://www.worldometers.info/world-population/population-by-country/.

[22] Chen Y., Li N., Lourenço J., Wang L., Cazelles B., Dong L., Li B., Liu Y., Jit M., Bosse N.I. (2022). Measuring the effects of COVID-19-related disruption on dengue transmission in southeast Asia and Latin America: A statistical modelling study. Lancet Infect. Dis..

[23] Khan S., Akbar S.M.F., Nishizono A. (2022). Co-existence of a pandemic (SARS-CoV-2) and an epidemic (Dengue virus) at some focal points in Southeast Asia: Pathogenic importance, preparedness, and strategy of tackling. Lancet Reg Health Southeast Asia.

[24] Prasertbun R., Mori H., Mahittikorn A., Siri S., Naito T. (2022). Pneumonia, influenza, and dengue cases decreased after the COVID-19 pandemic in Thailand. Trop. Med. Health.

[25] Mamun M.A., Misti J.M., Griffiths M.D., Gozal D. (2019). The dengue epidemic in Bangladesh: Risk factors and actionable items. Lancet.

[26] Dengue Outbreak No Mechanism to Collect Data from All Hospitals. NEWAGE Bangladesh..

[27] Rahmandad H., Lim T.Y., Sterman J. (2021). Behavioral dynamics of COVID-19: Estimating underreporting, multiple waves, and adherence fatigue across 92 nations. Syst. Dyn. Rev..

[28] World Health Organization (WHO) Dengue Situation Updates (2022). WHO Regional Office for the Western Pacific. https://apps.who.int/iris/handle/10665/352792.

[29] Lusting Y., Keler S., Kolodny R., Ben-Tal N., Atias-Varon D., Shlush E., Gerlic M., Munitz A., Doolman R., Asraf K. (2020). Potential antigenic cross-reactivity between SARS-CoV-2 and Dengue viruses. Clin. Infect. Dis..

[30] Ayala-Nuñez N.V., Jarupathirun P., Kaptein S.J., Neyts J., Smit J.M. (2013). Antibody-dependent enhancement of dengue virus infection is inhibited by SA-17, a doxorubicin derivative. Antivir. Res..

[31] Liu Y., Soh W.T., Kishikawa J.I., Hirose M., Nakayama E.E., Li S., Sasai M., Suzuki T., Tada A., Arakawa A. (2021). An infectivity-enhancing site on the SARS-CoV-2 spike protein targeted by antibodies. Cell.

