# Supplementary material for: Dengue Infections during COVID-19 Period: Reflection of Reality or Elusive Data Due to Effect of Pandemic
Source: Int J Environ Res Public Health. 2022 Aug 29;19(17):10768. doi: 10.3390/ijerph191710768 (PMC9518125; doi:10.3390/ijerph191710768)
Supplement: Supplementary file 1 [file ijerph-19-10768-s001.zip › ijerph-1835167-supplementary.pdf]

International Journal of
Environmental Research and Public Health

MDPI

Supplementary Data

Supplementary Table S1. COVID-19 and dengue cases in the Asian and Latin American countries during the COVID-19 pandemic period (2020~2021).

Region/Country	COVID-19 Cases		Dengue Cases	
	**2020**	**2021**	**2020**	**2021**
Asian Countries (Total)	**12,614,453**	**40,666,985**	**578,094**	**560,577**
India	10,286,329	24,575,250	44,585	193,245
Indonesia	743,198	3,519,522	95,971	71,796
Bangladesh	513,510	1,072,029	1405	28,429
Philippines	474,055	2,369,814	90,135	79,872
Nepal	260,593	564,838	428	365
Pakistan	113,884	330,944	6016	52,894
Malaysia	113,010	2,645,076	88,845	25,794
Singapore	58,599	220,806	35,315	5116
Sri Lanka	42,702	544,044	31,162	24,942
Thailand	6884	2,216,551	50,911	7180
Vietnam	1689	2,608,111	133,321	70,944
Latin American Countries (Total)	**14,136,813**	**27,690,192**	**2,217,728**	**1,227,652**
Brazil	7,718,847	14,568,674	1,467,142	975,474
Colombia	1,642,775	3,514,665	78,979	53,334
Argentina	1,625,514	4,028,894	59,358	3972
Mexico	1,413,935	2,555,751	120,639	36,742
Peru	1,015,137	1,281,694	563,94	49,274
Ecuador	212,512	338,741	16,570	20,592
Bolivia	158,372	441,381	109,010	8947
Honduras	121,827	257,625	25,180	19,753
Venezuela	113,884	330,944	6721	5926
Paraguay	107,964	360,382	223,782	16,897
Nicaragua	6046	11,441	53,953	36,741

Supplementary Table S2. The number of dengue cases may observe during the ongoing pandemic year (2022) in the Asian and Latin American countries.

Region/Country	Reported Average Cases between 2015~2021	Predicted Cases in 2022 *	Possible Chance % (Increase or Decrease)
Latin American Countries			
Brazil	1,194,102	1,181,121	−1.09
Mexico	134,859	79,310	−41.19
Nicaragua	76,873	78,971	+2.73
Colombia	75,348	65,186	−13.49
Paraguay	60,793	83,914	+38.03
Peru	39,077	43,422	+11.12
Honduras	36,861	44,879	+21.75
Bolivia	30,628	46,276	+51.09
Argentina	21,895	16,204	−25.99
Venezuela	19,974	10,837	−45.74
Ecuador	16,596	7628	−54.04
Asian Countries			
Philippines	207,353	152,753	−26.33
Vietnam	159,487	153,369	−3.83
India	130,545	141,938	+8.73
Indonesia	117,491	64,678	−44.95
Malaysia	89,769	52,929	−41.04
Thailand	84,023	26,087	−68.95
Sri Lanka	69,120	48,615	−29.67
Bangladesh	21,904	45,486	+107.66
Pakistan	21,330	42,862	+109.14
Singapore	12,409	18,002	+45.08
Nepal	3338	5392	+61.50

* Predicted dengue cases calculated based on the reported yearly cases between 2015~2021
"+" indicated the possibility of an increase in dengue cases; "–" indicated the possibility decrease in dengue cases
